# Evaluation and Comparison of the Pathogenicity and Host Immune Responses Induced by a G2b Taiwan Porcine Epidemic Diarrhea Virus (Strain Pintung 52) and Its Highly Cell-Culture Passaged Strain in Conventional 5-Week-Old Pigs

**DOI:** 10.3390/v9050121

**Published:** 2017-05-19

**Authors:** Yen-Chen Chang, Chi-Fei Kao, Chia-Yu Chang, Chian-Ren Jeng, Pei-Shiue Tsai, Victor Fei Pang, Hue-Ying Chiou, Ju-Yi Peng, Ivan-Chen Cheng, Hui-Wen Chang

**Affiliations:** 1 Graduate Institute of Molecular and Comparative Pathobiology, School of Veterinary Medicine, National Taiwan University, No. 1, Section 4, Roosevelt Rd., Taipei 10617, Taiwan; chyenjean@hotmail.com (Y.-C.C.); fei81005@gmail.com (C.-F.K.); f04644007@ntu.edu.tw (C.-Y.C.); crjeng@ntu.edu.tw (C.-R.J.); pang@ntu.edu.tw (V.F.P.); rudy4122@gmail.com (J.-Y.P.); Ivancheng@ntu.edu.tw (I.-C.C.); 2 School of Veterinary Medicine, National Taiwan University, No. 1, Section 4, Roosevelt Rd., Taipei 10617, Taiwan; psjasontsai@ntu.edu.tw; 3 Graduate Institute of Veterinary Pathobiology, College of Veterinary Medicine, National Chung Hsing University, 250 Kuo Kuang Rd., Taichung 402, Taiwan; hic01.chiou@gmail.com

**Keywords:** cell culture-adapted virus, conventional pig, neutralizing antibody, porcine epidemic diarrhea virus, viral shedding

## Abstract

A genogroup 2b (G2b) porcine epidemic diarrhea virus (PEDV) Taiwan Pintung 52 (PEDVPT) strain was isolated in 2014. The pathogenicity and host antibody responses elicited by low-passage (passage 5; PEDVPT-P5) and high-passage (passage 96; PEDVPT-P96) PEDVPT strains were compared in post-weaning PEDV-seronegative pigs by oral inoculation. PEDVPT-P5-inoculation induced typical diarrhea during 1–9 days post inoculation with fecal viral shedding persisting for 26 days. Compared to PEDVPT-P5, PEDVPT-P96 inoculation induced none-to-mild diarrhea and lower, delayed fecal viral shedding. Although PEDVPT-P96 elicited slightly lower neutralizing antibodies and PEDV-specific immunoglobulin G (IgG) and immunoglobulin A (IgA) titers, a reduction in pathogenicity and viral shedding of the subsequent challenge with PEDVPT-P5 were noted in both PEDVPT-P5- and PEDVPT-P96-inoculated pigs. Alignment and comparison of full-length sequences of PEDVPT-P5 and PEDVPT-P96 revealed 23 nucleotide changes and resultant 19 amino acid substitutions in non-structure proteins 2, 3, 4, 9, 14, 15, spike, open reading frame 3 (ORF3), and membrane proteins with no detectable deletion or insertion. The present study confirmed the pathogenicity of the PEDVPT isolate in conventional post-weaning pigs. Moreover, data regarding viral attenuation and potency of induced antibodies against PEDVPT-P5 identified PEDVPT-P96 as a potential live-attenuated vaccine candidate.

## 1. Introduction

Porcine epidemic diarrhea (PED) is caused by the PED virus (PEDV), which belongs to the *Alphacoronavirus* genus of the family *Coronaviridae*. PEDV contains a positive-sense, single-stranded RNA genome of approximately 28 kb in size, which encodes four structural proteins, namely, spike (S), envelope (E), membrane (M), and nucleocapsid (N) proteins as well as three non-structural proteins (NSP), namely, replicases 1a and 1b and open reading frame 3 (ORF3). The first outbreak of PED was recognized in England in 1971. During the 1980s and 1990s, PED caused severe economic losses in the swine industry in Europe and Asia, but it later turned into a sporadic disease [1]. However, since late 2010, devastating outbreaks of PED that affect all pigs, regardless of their age, with a mortality rate of up to 95% among suckling pigs, regardless of their vaccination status, have been reported in China [2]. The disease has rapidly spread to Asia and, subsequently, to North America, and has resulted in the death of millions of pigs, and has severely affected the swine industry [3,4].

Compared to traditional and CV777-based vaccine strains, the high genetic diversity of the genogroup 2b (G2b) PEDV suggests that PEDV CV777- or DR13-based attenuated vaccines may not completely protect pigs against the infection or control the disease progression [2,5]. Therefore, a new generation of PEDV vaccine against G2b PEDV is urgently required. In the past, successful attenuation of traditional PEDV strains such as SM98 [6], 83P-5 [3], KPED-9 [7], and DR13 [8] by in vitro serial passages in Vero cells has been achieved [9]. It has been demonstrated that conventional 11-day-old pigs orally inoculated with 2.55 × 10^5^ fluorescent focus forming units of the highly-passaged cell culture strain CV777 presents transient or no fecal viral shedding without clinical symptoms [10]. In addition, sows intramuscularly immunized with 10^7^ 50% tissue culture infective dose (TCID_50_)/mL of the highly cell culture-adapted CV777 strain twice every two weeks induces serum and colostrum antibody titers and reduces the mortality rate from 100 to 20% in neonatal piglets born to these vaccinated sows challenged with 10 50% lethal dose (LD_50_) of wild PEDV compared with those born to unvaccinated sows [7]. By using the cell culture passage strategy, evident attenuations of a high-passage PEDV YN Chinese strain (passage 144; YN-144) in 10-day-old conventional pigs [11] or highly-passaged PC21A-derived strains (passage 120 and passage 160) in 4-day-old, caesarian-derived, colostrum-deprived (CD/CD) or conventional suckling piglets [12] have been established. However, the detailed immune responses or the efficacy of cross-protection induced by these attenuated G2b PEDV strains against the parental virus or heterologous viruses are either unknown or only partially effective [12].

In Taiwan, new strains of PEDV have been detected since late 2013. Phylogenetic analysis of these field strains indicate that their *S* gene sequences are highly identical to each other and are closely related to those of G2b PEDV strains [5,13]. In the present study, a Taiwan PEDV Pingtung 52 (PEDVPT) strain was successfully isolated from a suckling pig during a farm outbreak in 2014 and serially passaged in Vero cells. To evaluate and compare the pathogenicity of the G2b Taiwan PEDVPT field isolate with its cell culture-adapted strain, we orally inoculated 5-week-old, PEDV-seronegative conventional pigs with a low-passage (passage 5; PEDVPT-P5) or high-passage (passage 96; PEDVPT-P96) PEDV strain. After confirming complete viral clearance in feces, these pigs were then challenged or re-challenged with PEDVPT-P5 to compare the efficacy of immune protection between the groups previously inoculated with PEDVPT-P5 or PEDVPT-P96.

## 2. Materials and Methods

### 2.1. Virus Isolation and Serial Passage of PEDVPT Strain

The parental PEDVPT strain (GenBank Accession No. KP276252) was isolated in early 2014 from the intestinal homogenate of a 7-day-old suckling pig in Taiwan and adapted to Vero cells as previously described [7,14]. Viral infection and propagation were confirmed by daily observation of cytopathic effects (CPE), real-time reverse transcription quantitative PCR (RT-qPCR), immunofluorescence assay (IFA), and immunocytochemistry (ICC) staining. In brief, Vero C1008 cells (American Type Culture Collection (ATCC) No. CRL-1586) were cultured overnight (O/N) to 80% confluence and were washed twice with modified post-inoculation medium (PI medium) containing Dulbecco’s modified Eagle’s medium (DMEM, Gibco, Grand Island, NY, USA) supplemented with tryptose phosphate broth (0.3%), yeast extract (0.02%), and 10 μg/mL of trypsin. After two blind passages, PEDV-inoculated Vero cells showing CPE, characterized by cell fusion, syncytial cell formation, and cell detachment, were subjected to three rounds of plaque purification. PEDV-infected Vero cells showing more than 90% CPE were subjected to one freeze-and-thaw cycle, and the supernatants were harvested. Viral stocks of the low-passage PEDV (passage 5; PEDVPT-P5) strain and the high-passage PEDV (passage 96; PEDVPT-P96) strain were prepared by serial passaging of the culture supernatants in Vero cells. The virus stocks were titrated by performing a 10-fold serial dilution of the supernatants in 96-well plates in triplicates for each dilution. The viral titers of the PEDVPT-P5 and PEDVPT-P96 stocks were 10^5^ and 10^6^ TCID_50_/mL, respectively.

### 2.2. Detection of PEDV Antigens by IFA and ICC

Supernatants of the virus-inoculated Vero cells showing typical CPE were eluted, 200 µL of 80% ice-cold acetone (Sigma-Aldrich, St. Louis, MO, USA) was added to each well, and the cells were fixed at −20 °C for 10 min. After fixation, acetone was removed, and the plate was air dried at room temperature for 30 min. The air-dried cells were then incubated with an in-house anti-PEDV N protein monoclonal antibody diluted 1000-fold (for ICC stain) or with PEDV hyperimmune pig serum diluted 200-fold (for IFA) and incubated at room temperature for 1 h. After incubation, the wells were washed three times with phosphate-buffered saline (PBS). For IFA, 100 μL of fluorescein isothiocyanate (FITC)-labeled goat anti-mouse immunoglobulin G (IgG) (Bethyl Laboratories, Montgomery, TX, USA) diluted to 200-fold in PBS was added to each well and incubated in dark at room temperature for 1 h. For ICC, a polyclonal anti-rabbit/mouse immunoglobulin EnVision-DAB+ system (Dako, Carpinteria, CA, USA) was used according to the manufacturer’s protocol. After incubation with the secondary antibody, each well was washed three times with PBS. For ICC staining, the labeled cells were exposed to 3,3′-diaminobenzidine (DAB) chromogen using a peroxidase DAB substrate kit (Dako, Carpinteria, CA, USA), according to the manufacturer’s instructions. For IFA, the labeled cells in the 96-well plate were mounted with 1% glycerol (Sigma-Aldrich) in PBS. The stained cells were detected using an inverted light/fluorescence microscope.

### 2.3. Comparative Pathogenicity and Assessment of Immunogenicity in PEDVPT-P5 and PEDVPT-P96-Inoculated 5-Week-Old Conventional Pigs

Eighteen 5-week-old, PEDV-seronegative, PEDV-fecal RNA-negative, Large White × Duroc crossbred pigs were selected from a conventional pig farm. Selected pigs were negative for porcine circovirus type II, porcine respiratory and reproductive virus, porcine rotavirus, transmissible gastroenteritis virus, porcine respiratory coronavirus, and porcine deltacoronavirus as tested by routine conventional or real-time PCR detection. These pigs were randomly assigned to four groups, viz., the mock-inoculated group with PEDVPT-P5 challenge (pigs A1 to A3; *n* = 3), mock-inoculated group without PEDVPT-P5 challenge (pigs A4 to A6; *n* = 3), the PEDVPT-P5-inoculated group with PEDVPT-P5 challenge (pigs B1 to B6; *n* = 6), and the PEDVPT-P96-inoculated group with PEDVPT-P5 challenge (pigs C1 to C6; *n* = 6). All pigs were labeled with ear tags. Three pigs of the same group were housed in a separate room. Each pig in the PEDVPT-P5- and PEDVPT-P96-inoculated groups was orally inoculated with 5 mL of 10^5^ TCID_50_/mL of the respective virus diluted in PI medium. Each pig in the mock-inoculated group received 5 mL of PI medium orally. Rectal swabs were collected every day to monitor the duration of viral shedding, and clinical signs were recorded and scored daily. To monitor seroconversion and mucosal immunity, 10 mL of ethylenediaminetetraacetic acid (EDTA)-anticoagulated blood and fecal swabs were collected from inoculated pigs every two weeks for detecting PEDV-specific plasma IgG and mucosal IgA, respectively. All experiments procedures performed on the animals were reviewed and approved by the Institutional Animal Care and Use Committee of National Taiwan University (Taipei, Taiwan, NTU105-EL-00087).

### 2.4. Re-Challenging with PEDVPT-P5 to Assess the Protection in PEDVPT-P5- and PEDVPT-P96-Inoculated Pigs

After confirming that all PEDVPT-P5- and PEDVPT-P96-inoculated pigs were seroconverted and negative for fecal virus shedding by RT-qPCR, all pigs were orally inoculated with 5 mL of 10^5^ TCID_50_/mL of PEDVPT-P5 to evaluate immune protection. At this time point, all pigs were 9-weeks old.

### 2.5. Scoring for Clinical Signs of Infection

After each inoculation, animals were monitored daily for clinical signs of infection until the end of the experiment. PEDV-associated diarrhea was scored based on the fecal consistency as follows: 0, normal; 1, loose; 2, semi-fluid; and 3, watery [15]. The weight of individual pigs in each group was monitored and recorded weekly.

### 2.6. RNA Extraction, cDNA Synthesis, and SYBR Green-Based Quantitative Real-Time PCR

Fecal swabs were diluted with 700 μL of PBS, and RNA was extracted using a QIAamp Viral RNA Mini Kit (Qiagen, Chatsworth, CA, USA), according to the manufacturer’s instructions. The total amount of feces contained in each fecal swab ranged from 0.25 to 0.35 g [15,16]. Complementary DNA (cDNA) synthesis was performed by reverse transcription using the QuantiTect Reverse Transcription Kit (Qiagen), according to the manufacturer’s instructions. The synthetized cDNA was amplified by real-time qPCR using SYBR Advantage qPCR Premix (Clontech, Palo Alto, CA, USA) under the following conditions: 45 cycles of 95 °C for 5 s, followed by 60 °C for 30 s. The primers used for detection of PEDV in the fecal samples have been previously published [17]. All real-time qPCR reactions were performed in duplicates, and the results were expressed as genomic equivalents (GE), as previously stated [17]. Ten-fold serial dilutions of a known amount of plasmid (pCR-XL-TOPO DNA; Thermo Fisher Scientific, Waltham, MA, USA) containing the PEDV *N* gene was used as the positive control to generate a standard curve. According to this standard curve, the detection limit of RT-qPCR was approximately 60 GE of DNA (data not shown). The amplification product for each reaction was also confirmed by performing the melting curve assay. Samples with a melting temperature of 84.7–86.1 °C were considered PEDV positive. The efficiency of the qPCR ranged from 90.42 to 96.92%.

### 2.7. Antibody Responses

For concentration and purification of PEDV virions, the cell component of the supernatants collected from PEDV-infected Vero cells was first freeze–thawed three times, and the cell debris was eluted by centrifugation at 6000× *g* for 30 min. To concentrate the virions, viral supernatants were pelleted by centrifugation at 75,000× *g* for 2.5 h using an Avanti J-25 centrifuge (Beckman, Fullerton, CA, USA) and were resuspended in PBS. Viral protein concentrations were determined using a Pierce BCA (bicinchoninic acid) Protein Assay Kit (Thermo Fisher Scientific Inc.). Ninety-six-well, flat-bottom plates (Corning Life Sciences, Corning, NY, USA) were coated O/N with 2 μg/well of concentrated PEDV virions. The PEDV-coated plates were washed six times with 100 mL of PBST (PBS containing 0.05% Tween 20), and wells were incubated for 1 h at room temperature with plasma samples diluted to 20-fold in PBS or with syringe-filtered (0.22 μm pore size) fecal samples, which was diluted two-fold in PBS; each fecal swab was diluted in 700 μL PBS. Each sample was added to the plate in duplicates. After incubation with samples, the wells were washed six times with PBST, and antibodies were detected using horseradish peroxidase (HRP) conjugated goat anti-pig IgG (Kirkegaard & Perry Laboratories, Gaithersburg, MD, USA) diluted to 1:1000 or goat-anti-pig IgA (Abcam, Cambridge, UK) diluted to 1:5000. After washing six times with PBST, 100 µL of tetramethylbenzidine (TMB) substrate solution (Kirkegaard & Perry Laboratories) was added to each well and incubated at room temperature for 20 min. The reaction was stopped using TMB stop solution (Kirkegaard & Perry Laboratories), and the optical density (OD) at 405 nm was recorded on an enzyme-linked immunosorbent assay (ELISA) reader. Antibody titers were expressed as sample-to-positive control ratio (S/P ratio) values. Using a panel of positive and negative controls, the cutoff values of plasma IgG and fecal IgA detection were 0.1 (95% confidence level) and 0.3 (95% confidence level), respectively.

### 2.8. Neutralizing Antibody Assay

For neutralization assay, 100 µL of Vero cell suspension at a density of 3 × 10^5^/mL were seeded onto 96-well culture plates (Corning Life Sciences) and incubated at 37 °C, 5% CO_2_ overnight to reach 70% confluence. Prior to performing neutralizing antibody assay, plasma samples were heated in water bath at 56 °C for 30 min to inactivate the complement and diluted at 1:10, 1:20, 1:40, 1:80, and 1:160 in PI medium. Fifty microliters of diluted plasma samples were admixed and incubated with 200 TCID_50_/mL PEDVPT-P5 at 37 °C and 5% CO_2_ for 1 h. After removing the medium from the 70% confluent Vero cells and washing the cells twice with PI medium, mixtures containing PEDVPT-P5 and different diluted plasma were added to Vero cells in duplicates and were incubated at 37 °C, 5% CO_2_ for 1 h. After 1 h of incubation, the supernatants were replaced by fresh PI medium. The cells were maintained at 37 °C and 5% CO_2_, and the CPE were monitored for the next three days. The neutralizing titer was determined as the last dilution without CPE.

### 2.9. Complete Viral Genome Sequencing and Full-Length Sequence Analysis

Viral RNA extraction and subsequent reverse transcription of both PEDVPT-P5 and PEDVPT-P96 were performed as previously described [5] using sequence-specific reverse primers (see Supplementary Table S1). The complete genomes of both viruses were obtained by using seven pairs of oligonucleotide primers that flank different regions of the novel strains of PEDV genome with at least 200-base pair overhangs (see Supplementary Table S1). The PCR amplicons of each fragment were gel-purified, cloned into plasmid vector (pJET1.2; Thermo Fisher Scientific), and subjected for sequencing after plasmid isolation. To acquire sequences of the extreme 3′ and 5′ ends, SMART RACE cDNA Amplification Kit was used according to the manufacture’s instruction (Clontech, Tokyo, Japan). Sequence assembly and comparison of complete genomes of both PEDVPT-P5 and PEDVPT-P96 were performed by using Lasergene 7.1 (DNASTAR, Madison, WI, USA) and MEGA7 [18] , respectively.

### 2.10. Statistical Analysis

The results of body weights, antibody titers, and fecal viral shedding were analyzed statistically using SAS 9.4 (Statistical Analysis System, SAS Institute Inc., Cary, NC, USA). The variables among groups were compared using one way analysis of variance (ANOVA) combined with Scheffe’s method. A *p*-value of <0.05 was interpreted as statistically significant.

## 3. Results

### 3.1. Viral Isolation of the PEDVPT Strain

**Figure 1:**
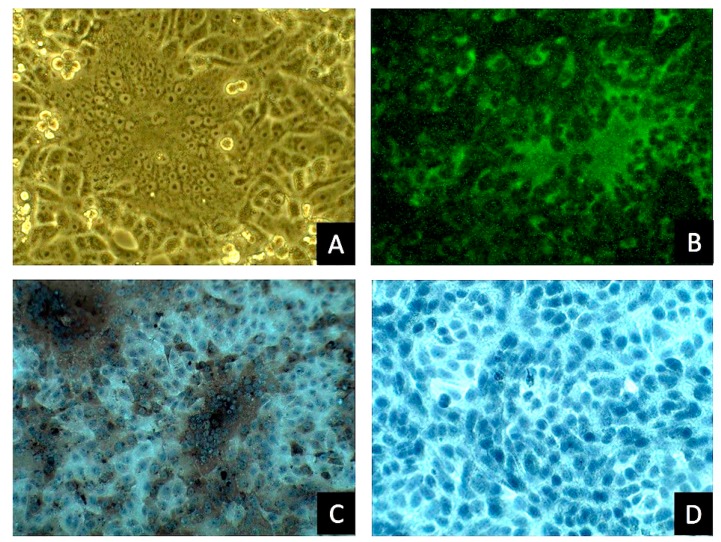
Detection of porcine epidemic diarrhea virus strain Pintung 52 (PEDVPT) infection in Vero cells. Typical PEDV-induced cytopathic effects (CPE) characterized by enlarged cells, cell fusion, and syncytia formation was present in cultures of PEDVPT passage 3-inoculated Vero cells (**A**). Positive signals located in the cytoplasm of typical syncytial cells were detected by immunofluorescence assay using PEDV hyperimmune pig serum (**B**) or by immunocytochemistry (ICC) using an anti-PEDV N protein monoclonal antibody (**C**). Non-PEDV infected Vero cells stained by ICC as negative control (**D**).

After two blind passages in Vero cells, the intestinal homogenate of inoculated cells at passage three showed typical PEDV-associated CPE [19] characterized by enlarged cells, cell fusion, and syncytial cell formation (Figure 1A). Positive, PEDV-specific cytoplasmic signals in cytopathic syncytial cells were detected by IFA using pig hyperimmune serum (Figure 1B) and by ICC using a monoclonal anti-PEDV N antibody (Figure 1C). No CPE and positive PEDV antigen signals were detected in Vero cells not inoculated with media containing PEDVPT (Figure 1D).

### 3.2. Pathogenicity of PEDVPT-P5 and PEDVPT-P96 in Conventional Pigs

The results of clinical scoring for all pigs are shown in Table 1. In the PEDVPT-P5-inoculated group, all pigs (6/6) presented with typical PEDV-associated, loose-to-watery diarrhea. With the exception of two pigs, most pigs in this group presented with loose diarrhea (score = 1) at 2–3 days post-inoculation (DPI) that worsened to watery diarrhea (score = 3) at 3–7 DPI and eventually ameliorated to loose diarrhea (score = 1) again at 6–9 DPI. Pig B5 exhibited only mild loose feces (score = 1) for three days during the study. Moreover, pig B6 presented with severe watery diarrhea (score = 3), severe dehydration, and electrolyte imbalance at 1 DPI. The animal was diagnosed with a poor prognosis and was immediately euthanized according to the guidelines of Institutional Animal Care and Use Committee of National Taiwan University (Taiwan, Republic of China). Most pigs in the PEDVPT-P96-inoculated group showed no obvious clinical signs of infection with the exception of two pigs (C3 and C4), which presented with transient loose feces (score = 1) at a few time points during the experiment. In the mock-infected groups, no clinical signs of infection were observed before challenge with PEDVPT-P5.

**Figure 2:**
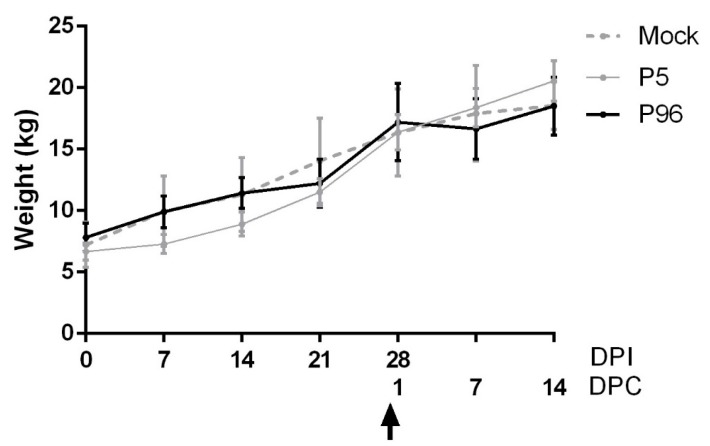
Weekly change in the body weight of 5-week-old pigs after porcine epidemic diarrhea virus strain Pintung 52 (PEDVPT)-P5 or PT-P96 inoculation. The weekly differences in mean body weight for each group are shown as mean ± standard error of the mean (SEM). The black, gray, and dashed lines illustrate the values obtained for the PEDVPT-P96-inoculated group, PEDVPT-P5-inoculated group, and mock-infected group, respectively. The arrow indicates the time point when the pigs were challenged with the low passage PEDVPT-P5 (27 DPI or 0 DPC) strain. P5: low passage virus, PEDVPT-P5; P96: high passage virus, PEDVPT-P96; mock: PI medium control; DPC: days post-challenge.

To evaluate the clinical effects of PEDV-associated diarrhea in post-weaning pigs, the mean value of the body weight in each group was also calculated (Figure 2). Although PEDVPT-P5-inoculated pigs showed a relative decrease in body weight gain compared to the other groups in the first two weeks post-inoculation, no significant differences in weight gain were observed among all groups in the study.

### 3.3. Fecal Viral Shedding in PEDVPT-P5- and PEDVPT-P96-Inoculated Pigs

**Figure 3:**
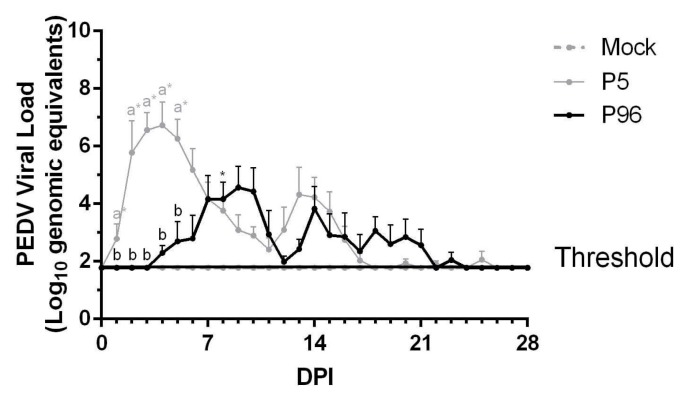
Fecal shedding of PEDV in 5-week-old pigs after porcine epidemic diarrhea virus strain Pintung 52 (PEDVPT)-P5 or PEDVPT-P96 inoculation. Changes in the mean values of genomic equivalents (GE)/mL are shown as log_10_ values ± SEM. The threshold indicates the limitation of detection for the SYBR Green-based real-time PCR (RT-PCR). The black, gray, and dashed lines illustrate the results obtained for the PEDVPT-P96-inoculated group, PEDVPT-P5-inoculated group, and mock-infected group, respectively. *: significant difference with the mock group (*p* < 0.05); a, b: significant difference between groups labeled with different alphabets (*p* < 0.05).

The changes and the length of fecal viral shedding in PEDVPT-P5- and PEDVPT-P96-inoculated pigs were determined using RT-qPCR and are presented as mean values in Figure 3. In the PEDVPT-P5-inoculated group (gray line in Figure 3), viral shedding was detected starting at 1 DPI, and it reached the peak viral load (6.73 log_10_ GE) at 4 DPI and was continuously detected until 17 DPI, after which intermittent low levels (1.93–2.07 log_10_ GE) were detected during 18–24 DPI. Fecal shedding in this group terminated at 26 DPI. The fecal viral shedding increased significantly (*p* < 0.05) in the PEDVPT-P5-inoculated group during the initial five DPI compared to the mock and the PEDVPT-P96-inoculated groups. Pigs in the PEDVPT-P96-inoculated group exhibited a delayed shedding pattern and lower peak viral loads compared to the PEDVPTP5-inoculated group. The average viral shedding in the PEDVPT-P96-inoculated group started at 4 DPI, exhibited a lower peak viral load (4.57 log_10_ GE) that was continuously detected until 21 DPI, after which intermittent viral shedding was observed until 22–23 DPI. Except the time point of 8 DPI, no significant difference was noted in fecal viral shedding between the mock and the PEDVPT-P96-inoculated groups during the study. No viral shedding in the mock-inoculated group was detected before challenged with PEDVPT-P5.

### 3.4. Antibody Responses Induced in Pigs after Inoculation with PEDVPT-P5 or PEDVPT-P96

**Figure 4:**
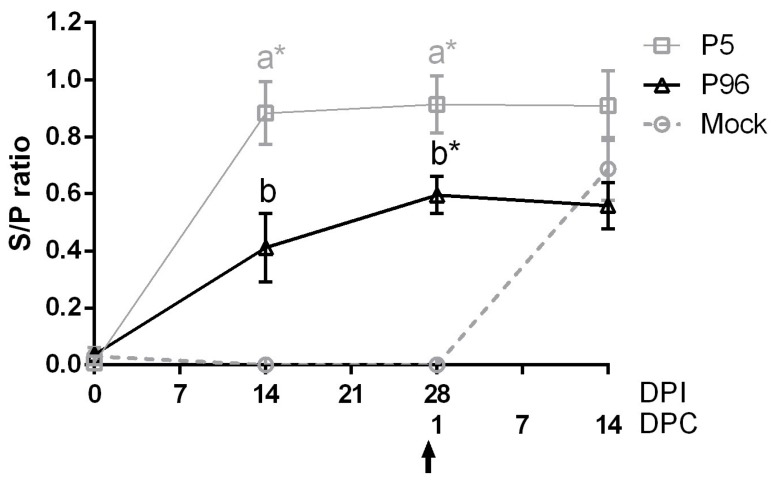
Changes in PEDV-specific immunoglobulin G (IgG) levels in plasma after PEDV strain Pintung 52 (PEDVPT)-P5 or PEDVPT-P96 inoculation and after PEDVPT-P5 challenge in all pigs. Plasma samples from ethylenediaminetetraacetic acid (EDTA)-anticoagulated blood were collected biweekly and diluted 20-fold. The levels of PEDV-specific IgG were detected by ELISA with PEDV particles as coating antigen and expressed as sample-to-positive control ratios (S/P ratio). S/P ratios were defined as the difference between the optical density (OD) values of sample and negative control divided by the difference between OD values of positive and negative controls. PEDV-specific plasma IgG levels are represented as mean ± SEM. The arrow indicates the time point when pigs were challenged with the low passage PEDVPT-P5 (27 DPI or 0 DPC) strain. The black, gray, and dashed lines illustrate the results for the PEDVPT-P96-inoculated group, PEDVPT-P5-inoculated group, and mock-infected group, respectively. *: significant difference with the mock group (*p* < 0.05); a, b: significant difference between groups labeled with different alphabets (*p* < 0.05).

**Figure 5:**
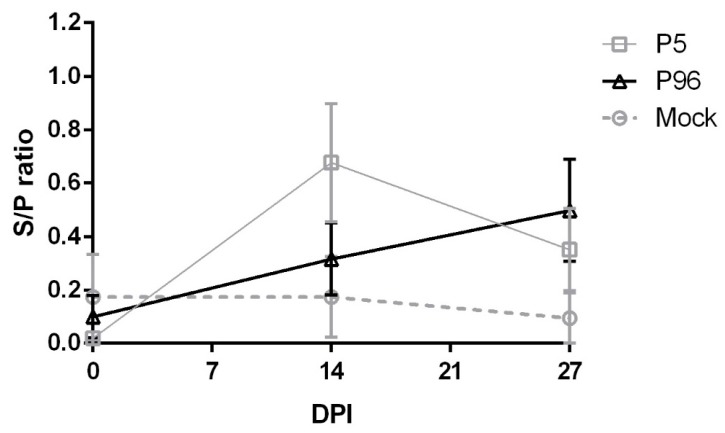
Changes in PEDV-specific mucosal IgA levels obtained from fecal swabs after PEDV strain Pintung 52 (PEDVPT)-P5 or PEDVPT-P96 inoculation of pigs. Each fecal swab was diluted with 700 μL PBS and filtered; the supernatants were diluted two-fold, and the levels of PEDV-specific IgA were detected by ELISA with PEDV virions as the coating antigen. The results obtained were expressed as sample-to-positive ratios (S/P ratio), which were defined as the difference between the OD values of sample and negative control divided by the difference between the OD values of positive and negative control, with SEM. The black, gray, and dashed lines illustrate the results of the PEDVPT-P96-inoculated group, PEDVPT-P5-inoculated group, and mock-infected group, respectively.

To evaluate the plasma IgG and fecal IgA antibody responses induced by PEDVPT-P5 and PEDVPT-P96 inoculations in conventional pigs, a whole PEDV particle-based ELISA was performed. The levels of plasma IgG (Figure 4) and fecal IgA (Figure 5) were measured in pigs at 0, 14, and 28 DPI. Elevated PEDV-specific IgG level was detected in all PEDVPT-P5- and PEDVPT-P96-inoculated pigs at 14 DPI, which reached a plateau at 28 DPI. In the PEDVPT-P96-inoculated group, PEDV-specific plasma IgG mean values (reported as the S/P ratio) ranged from 0.41 to 0.60. These values were significantly lower than those of the PEDVPT-P5-inoculated pigs, which ranged from 0.88 to 0.91 at 14–28 DPI, and were significantly higher than those of the mock-inoculated groups at 28 DPI. As for mock-infected pigs, no seroconversion was detected before the challenge with PEDVPT-P5 at 28 DPI (Figure 4). For the induction of mucosal IgA (Figure 5), a weak but detectable elevation of the mean S/P ratios for PEDV-specific IgA (0.31) was observed in both PEDVPT-P5 and PEDVPT-P96-inoculated pigs as early as at 14 DPI. No detectable mucosal IgA was noted in the mock-infected group during the study.

### 3.5. Neutralizing Antibodies Detection

**Figure 6:**
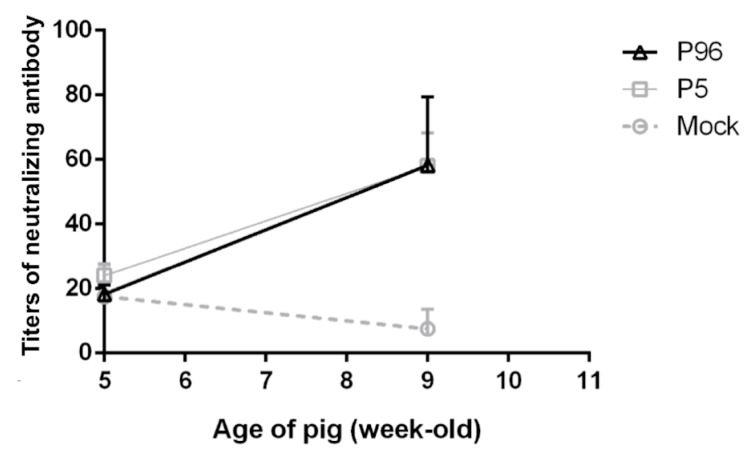
Changes in plasma PEDV neutralizing antibody levels after inoculation with PEDV strain Pintung 52 (PEDVPT)-P5 or PEDVPT-P96. The plasma samples were collected from 5- and 9-week-old (0 and 27 DPI) pigs, and neutralizing antibody assay was performed. Values are represented as mean ± SEM. The black, gray, and dashed lines illustrate the results of the PEDVPT-P96-inoculated group, PEDVPT-P5-inoculated group, and mock-infected group, respectively.

To evaluate the neutralizing antibody titer, PEDVPT-P5 was used as the challenging virus for the neutralization assay. The mean values of PEDV-specific neutralizing antibody titers in PEDVPT-P5- and PEDVPT-P96-inoculated pigs at 0 and 27 DPI are presented in Figure 6. The neutralizing antibody titer in all animals harbored a background titer of <20-fold at 0 DPI. Elevations in the mean value of neutralizing antibody titers in PEDVPT-P5- and PEDVPT-P96-inoculated pigs at 27 DPI ranged from 24- to 58-fold and from 18.3- to 58.3-fold, respectively, and were relatively higher (although not statistically significant) than those of mock-inoculated pigs, which ranged from 17.5 to 7.5-fold.

### 3.6. Immune Protective Efficacy Induced in Pigs after PEDVPT-P5 or PEDVPT-P96 Inoculation

**Figure 7:**
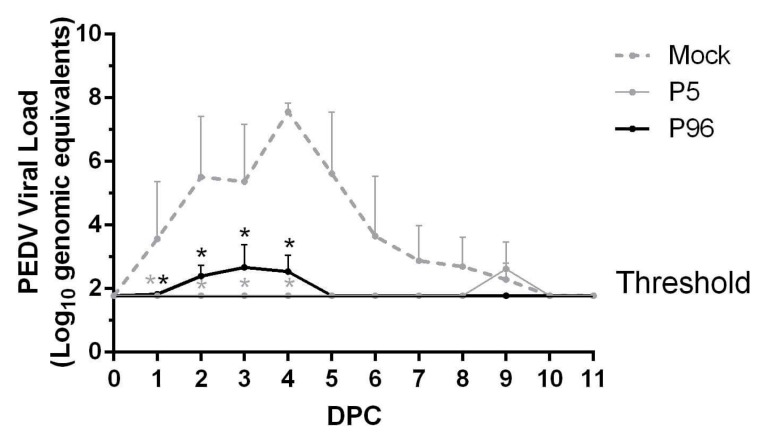
Fecal shedding of porcine epidemic diarrhea virus (PEDV) in PEDV strain Pintung 52 (PEDVPT)-P5- or PEDVPT-P96-inoculated pigs following PEDVPT-P5 challenge. Changes in the mean values GE/mL are shown as log_10_ values ± SEM. The threshold indicates the limitation of detection by the SYBR Green-based RT-PCR. The black, gray, and dashed lines illustrate the results for the PEDVPT-P96-inoculated group, PEDVPT-P5-inoculated group, and mock-infected group, respectively. *: significant difference in the mock group (*p* < 0.05).

To evaluate whether PEDVPT-P5 and PEDVPT-P96 inoculation induced immune protection against PEDVPT-P5, three pigs (A1–A3) in the mock-infected group and all pigs in both PEDVPT-P5- and PEDVPT-P96-inoculated groups were challenged with 5 mL of 10^5^ TCID_50_/mL of PEDVPT-P5 at 27 DPI. The three pigs in the mock group (A1–A3) challenged with PEDVPT-P5 started to develop loose diarrhea at 2 DPC that worsened to watery diarrhea at 3–5 DPC and terminated or ameliorated to loose diarrhea at 5–7 DPC (Table 1). Fecal viral shedding (Figure 7) was detected in these pigs starting at 1 DPC with a mean peak value of 7.56 log_10_ GE at 4 DPC and was continuously detected until 9 DPC. Furthermore, an elevated level of PEDV-specific plasma IgG (mean S/P ratio = 0.69) was detected in all three pigs (A1–A3) at 14 DPC (Figure 4). The clinical signs of infection, fecal viral shedding, and plasma IgG values of the other three pigs in mock group (A4–A6) not challenged remained undetectable during the experiment.

In PEDVPT-P5-inoculated pigs, no diarrhea was observed after the subsequent PEDVPT-P5 challenge, but a single time point of low-level of fecal viral shedding was detected at 9 DPC. In the PEDVPT-P96-inoculated group, three out of six pigs showed clinical loose feces (score = 1) at one time point and a transient detectable fecal viral shedding of 1.83–2.67 log_10_ GE during 1–4 DPC, whereas the other three pigs in the group showed no clinical signs of infection during 11 days of monitoring after PEDVPT-P5 challenge (Table 1). In all PEDVPT-P96- and PEDVPT-P5-inoculated pigs, no detectable increases in the mean values for plasma PEDV-specific IgG S/P ratios were noted after the challenge.

### 3.7. Sequence Comparison of PEDVPT-P5 and PEDVPT-P96 Strains

To gain a deeper understanding of the potential molecular mechanisms of viral attenuation of the high passage PEDVPT-P96 strain, we performed full-length sequencing of both PEDVPT-P5 and PEDVPT-P96 strains, and the sequence data were deposited in GenBank under accession Nos. KY929405 and KY929406, respectively. The complete genome sequence of both PEDVPT-P5 and PEDVPT-P96 were 28,038 nucleotides (nt) in length, excluding the 3′ poly(A) tail. After sequence alignment, no deletion or insertion was detected on comparing PEDVPT-P5 with PEDVPT-P96. In total, 23 nucleotide changes and the resultant 19 amino acid (aa) substitutions were revealed (Table 2). The *S* and *M* genes appeared to be the most variable, and each contained nine (T144I, F554S, S887R, S968A, I1021S, R1026K, L1252R, C1354F, C1358F) and three (I12V, S79A, F145L) aa changes, respectively. In the *S* gene, most mutations (7/9) were located in the S2 region. Moreover, many mutations were notably found in the functional regions, viz., the F554S in the CO–26K equivalent neutralizing epitope (COE) and C1354F as well as C1358F in the transmembrane domain. Among other genes encoding structural proteins, both the *E* and *N* genes were highly conserved and they retained 100% amino acid homology between these two viruses. For NSP, nine nucleotide changes were found in the *ORF1a/b* gene, but only five of these mutations resulted in aa substitutions, located at *NSP2* (K159N and T510I), *NSP3* (F669S), *NSP4* (E421A), and *NSP15* (M252I) genes. The *ORF3* gene also harbored one aa substitution (Y170H).

## 4. Discussion

In the present study, we successfully isolated and characterized the pathogenicity of a new Taiwan PEDVPT strain, PEDVPT-P5. We have demonstrated that this G2b PEDVPT-P5 strain can induce typical watery diarrhea in 5-week-old pigs but milder symptoms and a shorter viral shedding period in 9-week-old pigs. By serial passaging of PEDVPT in Vero cells, a PEDVPT-P96 viral stock was also prepared. Compared to PEDVPT-P5-inoculated pigs, PEDVPT-P96-inoculated pigs showed a delayed, mild, and transient diarrhea, or in some cases undetectable, providing evidence of viral attenuation. Of importance, inoculation of both PEDVPT-P5 and PEDVPT-P96 induced PEDV-specific plasma IgG, mucosal IgA, and neutralizing antibodies and elicited protection against a subsequent challenge with the low passage PEDVPT-P5 strain. This study provides valuable information regarding the viral shedding period, immunogenicity, and pathogenicity of different passaged PEDV in conventional post-weaning pigs. Moreover, the evidence of viral attenuation and the efficacy of protection induced by PEDVPT-P96 in conventional pigs highlights PEDVPT-P96 as a potential, live-attenuated PEDV vaccine candidate.

It has been shown that PEDV can infect seronegative pigs of all ages. However, the susceptibility to PEDV infection is age-dependent and is significantly higher in neonatal pigs than in weaning pigs [20]. In the present study, we demonstrated that compared to 5-week-old pigs, which exhibited PEDV-associated clinical signs for 7–9 days and long viral shedding period of 26 days, older 9-week-old pigs exhibited milder clinical symptoms for a shorter period lasting of 4–7 days and viral shedding lasting for only nine days. Furthermore, we demonstrated that even without displaying clinical signs, the viral shedding periods could be longer than three weeks in PEDV-infected post-weaning pigs. It has been reported that oral fluid samples are suitable for screening PEDV in commercial growing pig herds [21]. These findings suggest that routine screening for oral and fecal PEDV shedding in clinically healthy pigs when purchasing feeder pigs from swine producers is necessary to reduce the risk of disease transmission from farm to farm.

In the present study, we demonstrated that 5-week-old conventional pigs inoculated with the PEDVPT-P5 strain exhibited fecal viral shedding, peaked at 2–4 DPI, and persisted for 26 days. An increase in PEDV-specific IgG titer in these pigs started at 14 DPI and reached a plateau at 21 DPI. The result is similar to a previous finding in 4-week-old conventional pigs challenged with 1.42 × 10^5^ TCID_50_ of a USA/KS/2013 PEDV isolate. In this study, USA/KS/2013 isolate-inoculated pigs displayed fecal viral shedding primarily during the first two weeks with a peak at 5–6 DPI and fecal swabs that were positive for PEDV nucleic acid at 21 and 28 DPI in some pigs. Seroconversion was also noted at 14 DPI and reached a peak level at 21 DPI [22]. When the spike sequence of the USA/KS/2013 isolate was compared to the Taiwan PEDVPT isolate, we found that these two viruses share high sequence identity (99%).

In Asia, the attenuated CV777-based and DR13-based PEDV vaccines have been used for decades and, as a consequence, PED in Asia has been generally under control with low prevalence and mortality rates in the past [9]. These live-attenuated vaccines are derived from cell culture-adapted PEDV strains and have proven to be effective in controlling PED by reducing the severity and duration of diarrhea, shortening the period of viral shedding, stimulating the immune response of sows, and protecting the delivered piglets through elevated maternal antibodies [7,23,24]. Unfortunately, the exposure to traditional PEDV strains or the above-mentioned live-attenuated vaccine strains provide limited cross-protection against the epidemics of the novel virulent PEDV strains [15]. Therefore, a novel vaccine against these new strains is urgently needed. In the present study, we have developed a high passage PEDVPT-P96 viral stock by serial passaging in Vero cells and have provided evidence of its viral attenuation. In addition, PEDVPT-P96 inoculation of pigs induced plasma IgG, mucosal IgA, and neutralizing antibodies and provided complete immunization against a subsequent low passage PEDVPT-P5 challenge, suggesting that PEDVPT-P96 may serve as a potential live-attenuated vaccine candidate.

By comparing the sequences of the parental virulent PEDVPT-P5 and attenuated PEDVPT-P96 strains to identify potential genetic determinants for viral attenuation, we demonstrated that most non-silent mutations are located in the *S* gene. On comparing the result of sequence analysis with other previous studies, two mutations (S887R and C1354F) identified in the attenuated late passages of YN1 strain were also identified in our PEDVPT-P96 strain. However, the deletion at the position of aa 144 of the *S* gene, suggested by the same study as a potential attenuation marker for PEDV [10] was not found in PEDVPT-P96; alternatively, an aa substitution (T144I) occurred in this particular region. The clinical significance of these mutations requires further elucidation. Of note, one unique aa substitution (F554S) was observed in the COE neutralizing domain in PEDVPT-P96, but the mechanism of how it interferes with antibody recognition remains to be elucidated. However, the results of in vivo animal challenge performed in this study suggest that this mutation does not result in the loss of the cross-protection against the parental PEDVPT-P5 strain. In the present study, aa substitutions of F669S in the *NSP3* gene and M252I in the *NSP15* gene were identified in PEDVPT-P96. The NSP of coronaviruses are well known to be responsible for viral replication, and many of them are reported to be interferon (IFN) antagonists. In PEDV, NSP1, NSP3, NSP7, NSP14, NSP15, and NSP16 have been demonstrated to suppress the IFN-β and IRF3 promoter activities [23]. The effects of mutations in *NSP3* and *NSP15* genes of PEDVPT-P96 on immune modulation need further investigation. Unlike other reports wherein the *NSP3* and *ORF3* genes tend to comprise more mutations through PEDV attenuation, only one non-silent mutation was observed in each gene in the present study. To date, many studies have been conducted aiming to map attenuating mutation(s) [10,11,24]; however, there is little consistency among different studies, indicating that different strains may harbor different mutation patterns, thereby suggesting that the attenuation may be multifactorial and introduction of attenuating mutation(s) may have implications in generating a safe live attenuated vaccine. This information is invaluable for further quick and rational development of effective and genetically stable vaccines via reverse genetics.

It has been reported that the gut–mammary secretory IgA axis is the most promising and effective pathway against enteric diseases, including PED. For PEDV vaccine development, it is crucial for neonatal piglets to receive sufficient maternal antibodies against PEDV via colostrum or milk from previously immunized sows [8,25]. Furthermore, the amount and the duration of maternal antibodies is closely related to the antibody titers of the immunized sows [9]. Immune protection against PEDV infection, including pathogenicity, immunogenicity, and fecal viral shedding, in nursing pigs born to PEDVPT-P96-inoculated sows as well as the potential for reversal of virulence of the PEDVPT-P96 strain by serial animal passages will be further evaluated.

## 5. Conclusions

In conclusion, our study describes a PEDV challenge model using conventional post-weaning pigs to simulate the actual immune situation in the field and provides a model for the preclinical evaluation of vaccines and other interventions aiming to prevent PEDV infection. A high passage PEDVPT-P96 strain has also been demonstrated as a potential live-attenuated PEDV vaccine candidate. The present study provides insights into the pathogenicity, length of viral shedding, and immunogenicity of different passages of cell-adapted PEDV in conventional pigs.

## Figures and Tables

**Table 1:** Clinical scoring of fecal consistency.

Age (Weeks)	5						6							7			8		9							10
DPI	1	2	3	4	5	6	7	8	9	10	11	12	13	14	15	16	17–21	22–27								
DPC																		0	1	2	3	4	5	6	7	8–11
Mock group																										
A1	0	0	0	0	0	0	0	0	0	0	0	0	0	0	0	0	0	0	0	1	3	3	3	2	1	0
A2	0	0	0	0	0	0	0	0	0	0	0	0	0	0	0	0	0	0	0	0	0	1	3	0	0	0
A3	0	0	0	0	0	0	0	0	0	0	0	0	0	0	0	0	0	0	0	0	2	3	0	0	0	0
A4	0	0	0	0	0	0	0	0	0	0	0	0	0	0	0	0	0	0	0	0	0	0	0	0	0	0
A5	0	0	0	0	0	0	0	0	0	0	0	0	0	0	0	0	0	0	0	0	0	0	0	0	0	0
A6	0	0	0	0	0	0	0	0	0	0	0	0	0	0	0	0	0	0	0	0	0	0	0	0	0	0
PEDVPT-P5 group																										
B1	0	1	1	2	3	3	1	0	0	0	0	0	0	0	0	0	0	0	0	0	0	0	0	0	0	0
B2	0	2	3	3	2	3	2	0	0	0	0	0	0	2	1	0	0	0	0	0	0	0	0	0	0	0
B3	0	0	2	3	3	3	2	0	0	0	0	0	0	0	0	0	0	0	0	0	0	0	0	0	0	0
B4	0	0	2	2	2	3	3	2	1	0	1	0	0	0	0	0	0	0	0	0	0	0	0	0	0	0
B5	0	0	1	1	0	1	0	0	0	0	0	0	0	0	0	0	0	0	0	0	0	0	0	0	0	0
B6	2 ^†^																									
PEDVPT-P96 group																										
C1	0	0	0	0	0	0	0	0	0	0	0	0	0	0	0	0	0	0	0	0	0	0	0	0	0	0
C2	0	0	0	0	0	0	0	0	0	0	0	0	0	0	0	0	0	0	0	0	0	0	0	0	0	0
C3	0	0	0	0	0	0	1	0	1	0	0	0	0	0	0	0	0	0	0	0	0	0	0	0	0	0
C4	0	0	0	0	0	0	0	0	0	0	0	0	0	0	0	1	0	0	0	1	0	0	0	0	0	0
C5	0	0	0	0	0	0	0	0	0	0	0	0	0	0	0	0	0	0	0	0	0	1	0	0	0	0
C6	0	0	0	0	0	0	0	0	0	0	0	0	0	0	0	0	0	0	0	0	0	1	0	0	0	0

The scores were graded as follows: 0, normal; 1, loose diarrhea; 2, semi-fluid diarrhea; and 3, watery diarrhea. Pigs of each group were orally inoculated with post-inoculation (PI) medium, porcine epidemic diarrhea virus strain Pintung 52 (PEDVPT)-P5, or PEDVPT-P96 at 5-weeks old and later re-challenged or challenged with PEDVPT-P5 at 9-weeks old (27 days after the first inoculation). DPI: days post-inoculation; DPC: days post-challenge; PEDVPT-P5: low passage PEDV strain; PEDVPT-P96: high passage PEDV; mock: PI medium control. † Euthanized due to severe clinical signs and poor prognosis.

**Table 2:** Changes in nucleotides and amino acid between PEDVPT-P5 and PEDVPT-P96.

Gene	Nucleotide Change			Amino Acid Substitution
	Position	PEDVPT-P5	PEDVPT-P96	
*NSP2*	1099	G	T	K159N
*NSP2*	2151	C	T	T510I
*NSP3*	3172	T	C	---
*NSP3*	4983	T	C	F669S
*NSP4*	8518	T	C	---
*NSP4*	9102	A	C	E421A
*NSP9*	11951	C	T	---
*NSP14*	17065	T	A	---
*NSP15*	19471	G	T	M252I
*S*	21064	C	T	T144I
*S*	22294	T	C	F554S
*S*	23292	A	C	S887R
*S*	23535	T	G	S968A
*S*	23695	T	G	I2021S
*S*	23710	A	G	R1026K
*S*	24388	T	G	L1252R
*S*	24694	G	T	C1354F
*S*	24706	G	T	C1358F
*ORF3*	25301	T	C	Y170H
*E*	25517	C	T	---
*M*	25720	A	G	I12V
*M*	25921	T	G	S79A
*M*	26119	T	C	F145L

---: silent mutation.

## Supplementary Materials

The following are available online at www.mdpi.com/1999-4915/9/5/121/s1, Table S1: List of sequencing primers.
