# Supplementary material for: Evaluation and Comparison of the Pathogenicity and Host Immune Responses Induced by a G2b Taiwan Porcine Epidemic Diarrhea Virus (Strain Pintung 52) and Its Highly Cell-Culture Passaged Strain in Conventional 5-Week-Old Pigs
Source: Viruses. 2017 May 19;9(5):121. doi: 10.3390/v9050121 (PMC5454433; doi:10.3390/v9050121)
Supplement: Supplementary file 1 [file viruses-09-00121-s001.doc]

| **Primer** | **Sequence (5’-3’)** | **Position** |
| --- | --- | --- |
| 5’ RACE R | GATTACGCCAAGCTTACGCCTGCCTGGACAGGAATCTGG | 1 - 2757 |
| PEDV1F | CTGTCCTCTAGTTCCTGGTT | 165 - 4655 |
| PEDV1R | CATCTACCAAGCCATCC |  |
| PEDV2F | GGTCTTAAGGTCTTTAATGTTGTTGG | 4397 - 7907 |
| PEDV2R | CAGCAACTATGAACAGACACAAAAACC |  |
| PEDV3F | ACCTTTAATGATTGTCGTATGC | 7778 - 13422 |
| PEDV3R | GAGTGCTGTCTTATGCTCCGTG |  |
| PEDV4F | TTACCGAGTATACTATGATGG | 12929 - 17986 |
| PEDV4R | TTATGGCATCACCAGAAGC |  |
| PEDV5F | TACTGTTGTTTCAAACATGC | 17830 - 21816 |
| PEDV5R | CAGTAGGAGGTAAAACAGCC |  |
| 2F | TAAGTTGCTAGTGCGTAATAATGAC | 20568 - 24854 |
| 2R | CAGACTTCGAGACATCTTTG |  |
| PEDS1466F | TTCTGAGTCACGAACAGCCA | 22115 - 26934 |
| PEDN557R | CTCCACGACCCTGGTTATTT |  |
| 3’ RACE F | GATTACGCCAAGCTTTGGCATTTCTACTACCTCGGAAC | 26597 - 28038 |

Supplementary data:

Table S1: List of sequencing primers
